# Does the inclusion of rare variants improve risk prediction?

**DOI:** 10.1186/1753-6561-8-S1-S94

**Published:** 2014-06-17

**Authors:** Erin Austin, Wei Pan, Xiaotong Shen

**Affiliations:** 1 Division of Biostatistics, School of Public Health, University of Minnesota, Minneapolis, MN 55455-0392, USA; 2 School of Statistics, University of Minnesota, Minneapolis, MN 55455, USA

## Abstract

Every known link between a genetic variant and blood pressure improves the understanding and potentially the risk assessment of related diseases such as hypertension. Genetic data have become increasingly comprehensive and available for an increasing number of samples. The availability of whole-genome sequencing data means that statistical genetic models must evolve to meet the challenge of using both rare variants (RVs) and common variants (CVs) to link previously unidentified genome loci to disease-related traits. Penalized regression has two features, variable selection and proportional coefficient shrinkage, that allow researchers to build models tailored to hypothesized characteristics of the genotype-phenotype map. The following work uses the Genetic Analysis Workshop 18 data to investigate the performance of a spectrum of penalized regressions using at first only CVs or only RVs to predict systolic blood pressure (SBP). Next, combinations of CVs and RVs are used to model SBP, and the impact on prediction is quantified. The study demonstrates that penalized regression improves blood pressure prediction for any combination of CVs and RVs compared with maximum likelihood estimation. More significantly, models using both types of variants provide better predictions of SBP than those using only CVs or only RVs. The predictive mean squared error was reduced by up to 11.5% when RVs were added to CV-only penalized regression models. Elastic net regression with equally weighted LASSO and ridge components, in particular, can use large numbers of single-nucleotide polymorphisms to improve prediction.

## Background

The potential number of lives affected by successful early identification of patients at high risk for hypertension has motivated researchers across a spectrum of fields. On the frontier of risk prediction is the identification of genetic variants linked to traits such as high blood pressure. Advancements in sequencing have fostered the identification of a growing number of loci related to blood pressure. One such study performed by the International Consortium for Blood Pressure Genome-Wide Association Studies identified 29 single-nucleotide polymorphisms (SNPs) related to systolic blood pressure (SBP) [1]. A second compelling study concluded that perhaps as many as hundreds of SNPs affect blood pressure; moreover, rare variants (RVs) (variants with minor allele frequencies [MAFs] less than 5%) in addition to novel common variants (CVs) (MAFs greater than 5%) are necessary to explain the relationship between allelic variants and blood pressure [2].

One promising tool that may be able to leverage risk information simultaneously in both CVs and RVs is penalized regression. The range of available penalties allows researchers to estimate models with a mixture of two desirable properties: variable selection and proportional shrinkage of regression coefficients. The following work systematically measured the advantages of the different types of penalized regression methods in the prediction of SBP using only CVs, only RV, or combinations of the two.

## Methods

### Data

The primary source for genotypic, phenotypic, and covariate data was Genetic Analysis Workshop 18 (GAW18) data files. GAW18 data is provided for approximately 1000 Mexican American individuals consisting of 20 pedigrees enriched for type 2 diabetes. The pedigrees contained between 21 and 76 individuals. The phenotype of interest was the SBP measure from the first time point. Genotype data for more than 8,000,000 genome locations was derived from sequencing data for all odd-numbered chromosomes, representing all sequencing data made available by GAW18. Approximately one-third of the variants were common. The analysis accounted for the covariates age, gender, smoking status, and antihypertensive medication.

The pairwise correlation structure resulting from either a family structure or a cryptic population structure was removed using an estimate of the variance-covariance matrix. We estimated the variance-covariance structure as a function of the identity-by-state (IBS) matrix calculated from all available genome-wide association study data. EMMAX software [3] was used to obtain our IBS matrix estimate. For IBS matrix convergence, it was necessary to exclude individuals missing more than 10% of genotypes (pre-imputation). Therefore, the final sample size for this study was 759.

### Model

Let *Y_i _*be the SBP value at the first examination for subject i=1,…,n and define $X_{ij}$ as subject *i*'s minor allele count (0,1, or 2) for SNP j=1,…,p. Covariate information for subject  i is notated by $X_{i,age}$ for age, $X_{i,gen}$ for gender, $X_{i,smoke}$ for smoking status, and $X_{i,med}$ for antihypertensive medication use. The effect of antihypertensive medication on blood pressure is not consistent across samples; thus, it is not ideal to include patients using this medication. However, removing patients who used treatment medication from a diabetes-enriched sample would have excluded a significant part of the GAW18 data. We chose to incorporate use of antihypertensive medication as a covariate to account for medication use while minimizing assumptions about its impact on SBP. We assumed the following model relates the genotypic data to the phenotype : Y=Xβ+ε, where ε~N(0, ∑). Here, $Y=Y_{n\times 1}$, a vector of the phenotype measurement for the *n *samples; $X=X_{nX\left(1+4+p\right)},$ the design matrix for the genotype and covariate data, including a column of ones for $\beta_{0}$ estimation; and *ε *is a n×1 vector of random errors. The vector of predicted phenotypes, Ŷ, is then equal to $X\hat{\beta },$ where $\hat{\beta }$ is the maximum likelihood estimate (MLE) of the coefficient vector, *β*. More specifically,

$\hat{\beta }={\left(X^{\prime }{\text{Σ}}^{-1}X\right)}^{-1}X^{\prime }{\text{Σ}}^{-1}Y={\left[{\left({\text{Σ}}^{-1/2}X\right)}^{\prime }\left({\text{Σ}}^{-1/2}X\right)\right]}^{-1}{\left({\text{Σ}}^{-1/2}X\right)}^{\prime }{\text{Σ}}^{-1/2}Y={\left[{\left(X^{*}\right)}^{\prime }\left(X^{*}\right)\right]}^{-1}{\left(X^{*}\right)}^{\prime }Y^{*}$,

where $Y^{*}=\overset{-1/2}{\sum }Y$ and $X^{*}=\overset{-1/2}{\sum }X$. Thus, we can decorrelate our samples by premultipling both *Y *and *X *by $\overset{-1/2}{\sum }$. Kang et al (2010) demonstrated that the variance-covariance matrix, $\hat{\text{Σ}}$, can be estimated effectively as a function of the IBS matrix [3]. Kang et al showed the effectiveness of their method on both seemingly unrelated samples and samples with a substantial population structure [3]. For Kang et al's method, ${\hat{\sum }}_{g}^{2}=\sigma_{g}^{2}K+\sigma_{r}^{2}I_{n},$ where genetic variance parameter, $\sigma_{g}^{2}=\text{residual}$ variance parameter, and $K=I\hat{B}S$. We decorrelated our samples using the ${\hat{\sum }}^{-1/2}$ derived with the Kang et al method. During preparation of the final manuscript, work appeared by Rakitsch et al (2013) using a similar method to correct for population structures in a penalized regression approach to multimarker association mapping [4]. The present investigation studied a model of the new vector of decorrelated phenotypes, Y*, as a function of the new genotype and covariate matrix, X*. To be clear, the model used in the current study is Y*=X*β+ε*, where $\varepsilon^{*}\sim N\left(0,\sigma^{2}I_{n}\right)$. Note:$\sigma^{2}\approx 1$.

We first consider the unpenalized regression model. MLE is asymptotically unbiased with fixed *p *as n→∞, but it may not be for a large *p*. One possible remedy is to introduce regularization or penalization on regression coefficients. We obtained predictions of Y ^* ^by first obtaining $\hat{\beta }$, then ${Ŷ}^{*}=X^{*}\hat{\beta }$. For penalized regression methods, $\hat{\beta }$ is found by maximizing a penalized log-likelihood [5]: $l\left(\beta \right)-\lambda P\left(\beta \right)$.

Candidate penalties that perform variable selection are LASSO [6], SCAD [7], and the truncated *L*_1_-penalty (TLP) [8]. LASSO regression is performed by applying the penalty $P\left(\beta \right)=\sum_{k=1}^{p}\left|\beta_{k}\right|$. The SCAD penalty, $P\left(\beta ,\lambda \right)$, replaces $\lambda P\left(\beta \right)$ with $dP\left(\beta ,\lambda \right)/d\beta =\sum_{k=1}^{p}\lambda sign\left(\beta_{k}\right)\left[I\left(\left|\beta_{k}\right|\leq \lambda \right)+{\left(a\lambda -\left|\beta_{k}\right|\right)}_{+}/\left(a-1\right)\lambda \cdot I\left(\left|\beta_{k}\right|>\lambda \right)\right]$ for a=3.7. TLP regression uses $P\left(\beta \right)=\sum_{k=1}^{p}\text{min}\left(\left|\beta_{k}\right|/\tau ,1\right)$, where τ>0 is a thresholding parameter, beyond which there is no further penalty. Regressions using these penalties are three methods to shrink many regression coefficient estimates to 0, effectively selecting a subset of SNPs to be used for prediction. The variable selection feature can be of particular value in genetics settings such as ours where the number of true causative variants is likely a small fraction of the considered SNPs. If instead of variable selection, it is advantageous to proportionally shrink all regression coefficients, a candidate penalized regression method is ridge regression [9]. Ridge regression uses the penalty $P\left(\beta \right)=\sum_{k=1}^{p}\beta_{k}^{2}$. Elastic net penalized regression [10] is a hybrid of the two approaches, with a penalty structure that is a mixture of the LASSO and ridge penalties controlled by a user-specified mixing parameter, *α*, which is restricted to 0[1]. The elastic net penalty [10] is $P\left(\beta \right)=\left(1-\alpha \right){∥\beta ∥}_{2}^{2}+\alpha {∥\beta ∥}_{1}$, where  α is selected to match the desired balance of variable selection and coefficient shrinkage.

### Implementation

We restricted our study to the top 1000 CV SNPs and top 1000 RV SNPs as identified by the marginal significance of a Kruskal-Wallis test of the minor allele counts and SBP values for the 759 samples. The real-data observations were randomly divided into equally sized training, tuning, and testing sets (*n *= 253 for each), and a sequence of models was then fit on the training set. The sequence was defined by incremental increases in both the penalty and penalty-specific parameters (e.g., α and *τ*). The sequence of penalty (and tuning parameter when applicable) values used to fit the models spanned a range comprehensive enough to allow identification of the values which optimized performance for SCAD, LASSO, elastic net, and ridge regression. The additional tuning parameter,  τ, used in TLP-penalized regression greatly increased the computational time; therefore, the number of  λ and  τ pairs considered was constrained. The TLP results presented here likely underestimate the true performance of this method. In all penalized regressions, the optimal penalty value was the one minimizing prediction error in the estimated tuning phenotypes when applying the regression coefficients estimated from the training model based on that penalty value.

Models were fit in a directed way based on the number and type of variants. First, we examined only the top 10, 100, and 1000 most significant CVs. Then we repeated the examination using only the top 10, 100, and 1000 RVs. Next, we added 1, 10, 100, and 1000 of the complementary type of variant to the model. For example, after fitting a model with only the top 10 CVs, four models were fit using these same 10 CV SNPs *and *the top 1, then top 10, then top 100, and finally the top 1000 RVs. The formal assessment of the regression methods was done by applying the training coefficients corresponding to the optimal penalty to the testing data. This process of randomly dividing the real data set into training, tuning, and testing sets and then investigating the predictive performance of penalized regression methods was repeated 100 times as a form of cross-validation. The regression approaches were compared using predictive mean squared error (PMSE). Define $PMSE=\sum_{i=1}^{n}{\left({Ŷ}_{i}^{*}-Y_{i}^{*}\right)}^{2}/n.$ OLS, SCAD, LASSO, elastic net, and ridge-regression estimates were generated using R packages glmnet [5] and ncvreg [11]. TLP estimates were obtained using FGSG: Feature Grouping and Selection Over an Undirected Graph in Matlab [12].

## Results

**Figure 1:**
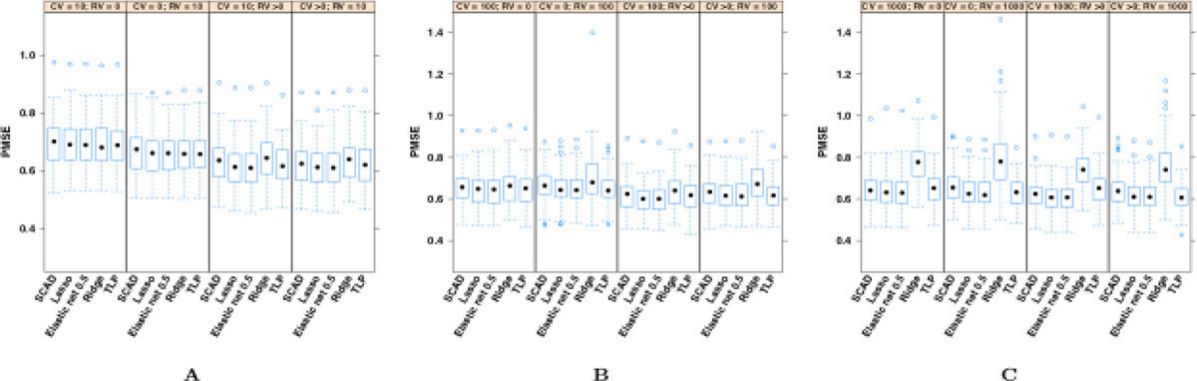
**Box plots of the median predicted mean square errors (PMSEs) calculated from the 100 randomly generated testing sets**. (A) Top 10 single-nucleotide polymorphisms (SNPs). (B) Top 100 SNPs. (C) Top 1000 SNPs. *CV*, common variant; *RV*, rare variant.

Descriptions of the PMSE of Y^* ^from the 100 randomly created testing data sets are presented in Figure 1 and Table 1. Figure 1 provides box plots for the PMSEs obtained using the different types of regression on the 100 data sets. The intent of Figure 1 is to provide an assessment of differences and reductions in PSME for different regression penalization methods within and between inputted SNP scenarios. Figure 1A presents results from models in which fitting was based on the top 10 SNPs for each of the variant types. Figure 1B presents results from models in which fitting was based on the top 100 SNPs for each of the variant types, and Figure 1C presents results from models in which fitting was based on the top 1000 SNPs for each of the variant types. In each figure, the first two columns represent models using only CVs or only RVs. The third column provides PMSEs of Y^* ^for the best model using the fixed number of CVs and 1, 10, 100, or 1000 RVs. For example, the column labeled CV = 10,RV>0 gives the smallest PSME from the four models using exactly the top 10 CVs and the top 1, 10, 100, or 1000 RVs. Similarly, the fourth column describes the model with the smallest PMSE using the fixed number of RVs and 1, 10, 100, or 1000 CVs. Figures 1A, 1B and 1C are plotted on the same scale to facilitate comparisons across them. Table 1 gives the median PMSE for the 12 modeling scenarios across the 100 data sets. Please note that the OLS PMSEs are not presented in Figure 1 because of their relative size.

**Table 1:** Median predicted mean square errors for calculated from the 100 randomly generated testing sets

	Top 10 SNPs				Top 100 SNPs				Top 1000 SNPs			
			
Regression method	CV only	RV only	CV = 10; RV >0	CV >0; RV = 10	CV only	RV only	CV = 100; RV >0	CV >0; RV = 100	CV only	RV only	CV = 1000; RV >0	CV >0; RV = 1000
OLS	3.723	0.719	1.856	0.722	107.775	24	98.109	21.748	370644.875	311336.868	37064.875	63274.28
SCAD	0.701	0.674	0.636	0.625	0.657	0.664	0.625	0.635	0.641	0.656	0.625	0.639
LASSO	0.691	0.661	0.613	0.612	0.649	0.644	0.601	0.616	0.632	0.625	0.608	0.611
Elastic net (α= 0.5)	0.689	0.661	0.610	0.610	0.646	0.643	0.601	0.613	0.630	0.619	0.608	0.610
Ridge	0.681	0.658	0.644	0.640	0.664	0.680	0.641	0.672	0.778	0.780	0.742	0.741
TLP	0.688	0.657	0.616	0.621	0.652	0.641	0.618	0.617	0.653	0.633	0.653	0.607

CV, common variant; RV, rare variant; SNP, single nucleotide polymorphism.

It is evident from Table 1 that penalized regression methods outperform OLS regardless of the number or type of candidate variants. Fixing the type of penalized regression and the number of top SNPs considered for the model allows us to uncover that RV-only models usually outperformed CV-only models. The difference was small, though. The central question to be answered by this work was whether adding RVs to CV models improved SBP prediction. We found that for penalized regression models, the inclusion of at least one of the complementary type of variant improved or maintained the performance of the model. This was true whether we fixed 10, 100, or 1000 top SNPs, added CVs to RV-only models, or added RVs to CV models. Again, the differences were small; however, small but perceptible shifts in the overall distributions as presented in Figure 1 support this conclusion.

Comparisons across models based on the top 10, top 100, and top 1000 SNPs revealed an interesting pattern. As the number of candidate SNPs increased, the sparse SCAD, LASSO, and TLP penalties were generally superior to the nonsparse ridge penalty. Differences were small, at most 0.1555 mm Hg, and need confirmation on different SBP real data sets. The conclusion should also be corroborated with simulated SBP data sets generated from genetic models reflecting a comprehensive range of possible SBP genetic architectures. Furthermore, although reductions in PMSE occurred within the same variant composition across the three top SNP groupings (e.g., comparing CV only for the top 10 with CV only with the top 100 SNPs), the gains were often less than those made just by adding the complementary type of variant to the model. Combined, these two results suggest that the true number of strong causative variants is at most moderate and includes both RVs and CVs. Ridge regression was the best or nearly identical to the best penalty choice when only the top 10 CVs or RVs were used, indicating that all of these top variants are integral in understanding the association between genotypes and SBP. TLP was a top performer with models using only the top 10 or top 100 RVs. As more SNPs of any type were included, the elastic net equally weighted to LASSO and ridge was generally superior. That is, there was a need for a selection element to distinguish noise from true effect, and there was a need for a nonsparse penalty feature to still incorporate larger numbers of SNPs in the regression model. This perhaps indicates that beyond a small set of strong causative SNPs, there are many SNPs that are truly associated with the outcome, but the majority of them have small marginal effects sizes. This could prove important when considering that previous research has found at least 29 causative SNPs; thus, undiscovered variants associated with SBP may have at most moderate effect sizes.

## Discussion

The strongest conclusion can be drawn about the effect of including RVs in addition to CVs when predicting SBP. The PMSE was reduced by up to 11.5%, and generally reduced between 4% and 9%, when RVs were added to CV-only penalized regression models. This was true when any of 10, 100, or 1000 top SNPs were used. PMSE comparisons of single-variant type models to combined-variant type models revealed that both RVs and CVs explain variance in SBP. Every penalty considered in the study improved SBP prediction over OLS. This was true whether estimation used only CVs, used only RVs, or used both types of variants. The elastic net penalized regression was best at leveraging the information in the additional SNPs (RVs or CVs) and produced the best overall models. (Again, the absolute reduction in PMSE was too small to be statistically significant because of the variance in the PSME median distributions.) Caution is needed when making conclusions about the TLP because of the limited number of combinations of λ and  τ studied because of time constraints. The results here likely understate the performance of TLP; thus, the small gains from using TLP with the top 10 and top 100 RVs warrant future analysis for possible confirmation. Work on the genotype-hypertension map should specifically consider RVs and CVs. The interesting result that a hybrid penalty with both selection and proportional shrinkage components performed best hints at an underlying architecture in which numerous SNPs with moderate main effects are interrelated in how they are associated with blood pressure. Overall, the results presented here provide evidence that penalized regression, especially a hybrid of LASSO and ridge regression, can be used to improve SBP prediction.

## Competing interests

The authors declare that they have no competing interests.

## Authors' contributions

WP designed the study framework, helped develop methodology, and helped rewrite the manuscript; EA performed the statistical analysis, drafted the manuscript, and helped rewrite the manuscript; and XS helped develop methodology and helped develop software used to conduct the analysis. All authors read and approved the final manuscript.
